# Fecal microbiota transplantation for chronic constipation: a systematic review and meta-analysis of clinical efficacy, safety, and microbial dynamics

**DOI:** 10.3389/fmicb.2025.1604571

**Published:** 2025-07-31

**Authors:** Ke Wang, Chun Gao, Li Zhu, Min Chen, Yi Xin Tong, Sheng Zhang

**Affiliations:** ^1^The Second Department of Surgery, Jiyuan Hospital of Traditional Chinese Medicine, Jiyuan, China; ^2^Department of Gastrointestinal Surgery, Tongji Medical College, Tongji Hospital, Huazhong University of Science and Technology, Wuhan, China

**Keywords:** fecal microbiota transplantation, chronic constipation, gut microbiome, microbiota remodeling, refractory constipation

## Abstract

**Background:**

Chronic constipation, a prevalent gastrointestinal disorder with limited treatment efficacy in refractory cases, has prompted exploration of fecal microbiota transplantation (FMT) as a novel therapeutic strategy. This systematic review and meta-analysis evaluate the efficacy, safety, and gut microbial dynamics of FMT in adults with chronic constipation.

**Methods:**

We systematically searched PubMed, Embase, Web of Science, and Cochrane Library up to January 2025, identifying 1,072 records. Nine studies (*n* = 245 patients) met inclusion criteria for qualitative synthesis, with eight contributing to meta-analysis. Outcomes included constipation remission and improvement, stool metrics, quality of life, and microbiota changes. Random-effects models analyzed pooled remission rates, mean differences (MDs), and heterogeneity (I^2^ statistics).

**Results:**

Fecal microbiota transplantation achieved a 50.7% pooled remission rate (95% CI: 38.7%–62.7%) and 64.8% improvement rate (95% CI: 51.4%–76.3%). Significant improvements were observed in stool consistency (MD = 1.32, 95% CI: 1.05–1.35), quality of life (GIQLI MD = 32.19, 95% CI: 17.15–47.23), and symptom severity (Wexner MD = −4.83, 95% CI: −7.15–2.51). Post-FMT microbiota analyses revealed enrichment of beneficial taxa (Bifidobacterium, Prevotella; Firmicutesacteroidetes) and suppression of pro-inflammatory Enterobacteriaceae. Transient gastrointestinal adverse events (e.g., bloating: 17.3%) resolved spontaneously, with no severe complications reported.

**Conclusion:**

Fecal microbiota transplantation demonstrates clinically meaningful symptom relief and gut microbiota remodeling in chronic constipation, supported by favorable short-term safety. While heterogeneity in protocols and limited RCT data warrant caution, these findings advocate standardized FMT frameworks and confirmatory trials to optimize therapeutics for refractory constipation.

**Systematic review registration:**

https://www.crd.york.ac.uk/prospero/, identifier CRD42025643634.

## Introduction

Chronic constipation, a prevalent functional gastrointestinal disorder affecting 10%–15% of adults globally, imposes substantial burdens on quality of life and healthcare systems. Its prevalence escalates with age, disproportionately impacting women (female-to-male ratio ∼2:1), and manifests as heterogeneous subtypes: slow-transit, outlet obstruction, or a combination thereof—as defined by Rome IV criteria. First-line therapies, including dietary fiber, osmotic laxatives, and prokinetic agents, fail to achieve sustained relief in 30%–40% of patients, underscoring an urgent need for mechanistically grounded interventions targeting underlying pathophysiology (Bharucha and Wald, 2019; Brenner et al., 2022; Drossman and Hasler, 2016; Harris and Chang, 2022; Wald, 2016).

Recent advances have delineated gut microbial dysbiosis as a pivotal contributor to chronic constipation. Comparative metagenomic analyses reveal consistent depletion of short-chain fatty acid (SCFA)-producing taxa (e.g., *Faecalibacterium prausnitzii*) and enrichment of methanogens (e.g., *Methanobrevibacter smithii*) in constipated individuals, disrupting colonic motility through impaired serotonin signaling, altered enteric nervous system activity, and methane-induced slowing of transit (Attaluri et al., 2010; Banaszak et al., 2023; Goya-Jorge et al., 2023; Huang et al., 2018). Germ-free rodent models transplanted with microbiota from constipated donors recapitulate delayed gut motility, while probiotic supplementation partially restores peristalsis, directly implicating microbial communities in disease pathogenesis (Ge et al., 2017; Peng et al., 2023; Roy and Dhaneshwar, 2023).

Fecal microbiota transplantation (FMT), which reconstitutes gut ecological balance through engraftment of donor-derived microbiota, has demonstrated transformative success in recurrent *Clostridioides difficile* infection (cure rates > 90%) and moderate efficacy in ulcerative colitis (Paramsothy et al., 2017; Walter and Shanahan, 2023). In functional gastrointestinal disorders, emerging evidence highlights FMT’s potential to modulate gut-brain axis signaling. A 2020 randomized controlled trial (RCT) reported significant improvements in stool frequency and abdominal pain in irritable bowel syndrome patients receiving FMT, mediated by restored microbial diversity and enhanced SCFA production (El-Salhy et al., 2020). Preliminary studies in chronic constipation suggest FMT may ameliorate symptoms by upregulating serotonin biosynthesis, reducing methane production, and reinforcing intestinal barrier integrity, yet current evidence remains limited to small, non-randomized cohorts (Li et al., 2024; Liu et al., 2021; Singh et al., 2022; Wang et al., 2024).

This systematic review and meta-analysis synthesize existing clinical data to rigorously evaluate FMT’s efficacy in adult chronic constipation, quantifying its effects on core endpoints (stool frequency, consistency) and patient-reported outcomes. By integrating microbial compositional changes with clinical responses, we aim to define actionable patterns linking microbiota remodeling to therapeutic success. Our findings will address critical gaps in evidence, inform future RCT design, and advance translational strategies leveraging the gut microbiome as a therapeutic target for chronic constipation.

## Materials and methods

### Protocol and registration

In this study, all procedures were conducted in accordance with the guidelines specified in the Preferred Reporting Items for Systematic Reviews and Meta-Analyses 2020 (PRISMA-2020) protocol (Page et al., 2021). The methodological approaches were consistent with the standards outlined in the Cochrane Handbook. (Higgins and Thomas, 2024) Additionally, this study has been registered with the International Prospective Register of Systematic Reviews, with the registration number PROSPERO: CRD42025643634.

### Search strategy and study selection

Up to January 2025, we conducted searches across four electronic databases: PubMed, Cochrane Library, Scopus, and Web of Science. We conducted our search using the following query to identify relevant studies on fecal microbiota transplantation: (“Fecal Microbiota Transplant*” OR FMT OR “Fecal Microbiota Transplant*” OR “microbiota transplant*” OR “microbiota transfer” OR “microbiome transfer” OR “microbiome transplant*” OR “feces transplant*” OR “Fecal Transplant*” OR “Fecal Transfer” OR “stool Transplant*” OR “stool Transfer” OR “stool therapy” OR “Donor Feces” OR “donor fecal” OR “donor stool” OR “fecal flora transplant*” OR “microflora transplant*” OR “Fecal therapy” OR “fecal bacteriotherapy” OR “feces bacteriotherapy” OR “rectal bacteriotherapy” OR “fecal flora bacteriotherapy” OR “fecal reconstitution” OR “flora reconstitution” OR “microbiome reconstitution” OR “feces reconstitution”) AND (“chronic constipation” OR “slow transit constipation” OR “constipation”). We only include studies in English in our search. Furthermore, we reviewed the reference lists of the included studies to identify additional relevant citations.

Titles and abstracts were independently screened by two reviewers, C.G. and M.C., to identify studies meeting the inclusion criteria and exclude irrelevant ones. Duplicate records were systematically removed, and reference lists of selected studies were manually searched to capture additional eligible studies. Discrepancies during the selection process were resolved through consultation with a third reviewer, Y.X.T., ensuring methodological rigor and decision consistency.

### Inclusion and exclusion criteria

Studies were considered for full review if they met the following criteria: single-arm or two-arm studies, retrospective studies, prospective studies, and randomized controlled trials, focusing on the effects of FMT on constipation. The population included adults with chronic constipation or slow transit constipation. Studies were excluded if they were abstracts, letters to the editor, conference proceedings, review articles, case reports, comments, or book chapters. Studies with insufficient data to summarize were also Additionally, a manual search of reference lists was conducted to identify potentially relevant articles for further study inclusion. Exclusion criteria comprised: (1) review articles, case reports, letters, comments, or book chapters; (2) studies with insufficient data to extract predefined efficacy endpoints (e.g., missing stool frequency metrics or symptom scores); (3) non-English publications.

### Bowel movement measurements and outcomes

The bowel movement measurement was evaluated by CSBM (complete weekly spontaneous bowel movements), CTT (colonic transit time), and BSFS (Bristol Stool Form Scale). Other evaluations included PAC-SYM (Patient Assessment of Constipation-Symptoms); PAC-QOL (Patient Assessment of Constipation Quality of Life); GIQLI (Gastrointestinal Quality-of-Life Index); SACS (Self-assessment of constipation symptoms). The primary outcome assessed in this systematic review was clinical remission rate, defined as the proportion of patients who had an average of three or more spontaneous complete bowel movements per week during the observation period. The secondary outcome was clinical improvement rate, defined as proportion of patients who were in remission or had an average increase of one or more CSBMs/week compared with baseline. Furthermore, the implementation of fecal microbial profiling during both pre- and post-transplantation phases was systematically recorded and critically appraised across all eligible studies in this systematic review.

### Data extraction and quality assessment

In this systematic review, data extraction was independently conducted by two reviewers using a pre-specified data extraction form. Key variables included first author, publication year, trial design, sample size, participant characteristics (e.g., constipation type), FMT intervention details (e.g., administration route), outcome measures (e.g., bowel movement frequency, symptom scores), and primary results (summarized in Table 1). Outcome data from intention-to-treat or per-protocol analyses were prioritized to ensure alignment with original study conclusions. To enhance reliability, the dual-reviewer system was implemented for cross-verification, with any discrepancies re-examined through iterative discussions until consensus was achieved. Additionally, the inclusion or omission of pre- and post-FMT fecal microbiome analysis in each study was explicitly documented to assess microbial dynamics in relation to clinical outcomes.

**TABLE 1:** Characteristics of studies included in systematic review.

References	Year		Patients’ characteristics	Study design	Sample size	Study endpoints	Follow-up and outcomes
Ge et al., 2016	2016	China	Slow transit constipation	Retrospective, Single-arm FMT group: via nasojejunal tube	21	CSBM, stool consistency (BSFS), GIQLI, PAC-SYM, CTT	12 weeks Remission rate: 42.9% Improvement rate: 66.7% Adverse event
Tian et al., 2016	2016	China	Slow transit constipation	Retrospective, Single-arm FMT group: via nasojejunal tube	24	CSBM, BSFS, Wexner score, GIQLI, CTT	12 weeks Remission rate: 37.5% Improvement rate: 50%
Tian et al., 2017)	2017	China	Slow transit constipation	RCT, FMT (via nasojejunal tube) vs. conventional therapy	49 (25/24)	CSBM, CTT, BSFS, Wexner score	12 weeks Remission rate: 36% vs 13.3% Improvement rate: 52% vs. 20% Adverse event
Ding et al., 2018	2018	China	Slow transit constipation	Propective, Single-arm FMT group: via nasojejunal tube	52	CSBM, BSFS, PAC-SYM, PAC-QOL	24 weeks Remission rate: 32.7% Improvement rate: 44.2% Adverse event
Tian et al., 2020	2020	China	Chronic functional constipation	Propective, Single-arm FMT group: via nasojejunal tube	34	Wexner score, SACS, BSFS, PAC-QOL, SDS, SAS	12 weeks Remission rate: 73.5% Improvement rate: 88.2% Adverse event
Zhang et al., 2021	2021	China	Refractory constipation	Retrospective, Single-arm FMT group: via nasojejunal tube	18	CSBM, stool consistency (BSFS), Wexner score, PAC-SYM, PAC-QOL	4 weeks Remission rate: 77.8% Microbime and metabolite change
Xie et al., 2021	2021	China	Slow transit constipation	Retrospective, Single-arm FMT group: via nasojejunal tube	8	CSBM, Wexner score, GIQLI, HAMD	6 weeks Remission rate: 62.5% Improvement rate: 75% Microbime and metabolite change Adverse event
Yang et al., 2021	2021	China	Mixed constipation	Retrospective, double-arm FMT group vs. Biofeedback group	85 (45/40)	CSBM, BSFS, PAC-SYM, GIQLI	48 weeks Mean CSBM: 3.61/week vs. 2.15/week (1 month); 3.25/week vs. 2.01/week (6 months); 2.71/week vs. 1.98/week (12 months) Microbime and metabolite change Adverse event
Wu et al., 2023	2023	China	Refractory functional constipation	Retrospective, Single-arm FMT group: colonscopy or nasojejunal tube	63	CSBM, Wexner score, BSFS score	24 weeks Remission rate: 50% Improvement rate: 73% Adverse event

FMT, fecal microbiome transplantation; CSBM, complete weekly spontaneous bowel movements; CTT, colonic transit time; BSFS, Bristol Stool Form Scale; PAC-SYM, Patient Assessment of Constipation-Symptoms; SBMs, spontaneous bowel movements; PAC-QOL, Patient Assessment of Constipation Quality of Life, GIQLI, Gastrointestinal Quality-of-Life Index; SACS, Self-assessment of constipation symptoms; SDS, Zung’s self-rating depression scale; SAS, Zung’s self-rating anxiety scale; HAMD, Hamilton Depression Scale.

Study quality was assessed using domain-specific tools: the Cochrane Risk of Bias 2.0 (RoB 2) tool for randomized trials and the Newcastle-Ottawa Scale (NOS) for observational studies. Two reviewers independently evaluated selection bias, performance bias, detection bias, attrition bias, and reporting bias for RCTs using RoB 2’s signaling questions. Observational studies were scored across NOS domains (selection, comparability, outcome) (Sterne et al., 2019; Wells et al., 2014). Discrepancies were resolved through consensus. The NOS evaluates studies based on eight criteria, encompassing three key domains: the selection of study participants, the comparability of the study’s design and analysis, and the assessment of study outcomes. Additionally, the level of certainty of the evidence presented in each study was assessed utilizing the Grading of Recommendations Assessment, Development, and Evaluation (GRADE) methodology (Guyatt et al., 2011)

### Statistical analysis and publication bias

A single-arm meta-analysis was conducted to estimate the pooled response rate of fecal microbiota transplantation (FMT) in chronic constipation. Proportions of responders (defined by study-specific criteria) were synthesized using a generalized linear mixed model (GLMM) with random effects, which accounts for between-study heterogeneity and stabilizes variance in sparse data through logit transformation. The GLMM framework was selected for its robustness in handling overdispersed binomial data and its alignment with recommendations for meta-analyses of proportions. Interstudy heterogeneity was quantified using the Cochran’s Q statistic (significance threshold *p* < 0.10) and the I^2^ statistic, interpreted as follows: I^2^ < 25% (low), 25%–50% (moderate), 50%–75% (substantial), and > 75% (considerable). To assess the robustness of pooled estimates, sensitivity analyses were performed by iteratively excluding individual studies. Potential sources of heterogeneity were qualitatively explored through examination of methodological variations in donor selection criteria, fecal processing protocols, and follow-up duration across studies.

A prediction interval (95% PI) was calculated to estimate the potential range of response rates in future studies. The analysis was performed using the metaprop function in the R package meta (v6.5-0). Forest plots were generated to visualize individual study proportions (with 95% confidence intervals), the pooled estimate, and prediction intervals. Weights assigned to each study were derived from the inverse variance method under the random-effects assumption. All statistical code was executed in R 4.3.1, with reproducibility ensured through script-based workflows. The GLMM models included a logit link function and restricted maximum likelihood (REML) estimation for variance components. Due to the absence of randomized controlled trials in the included studies, formal risk of bias assessment was not applicable.

## Results

### Search results and characteristics of included studies

**FIGURE 1:**
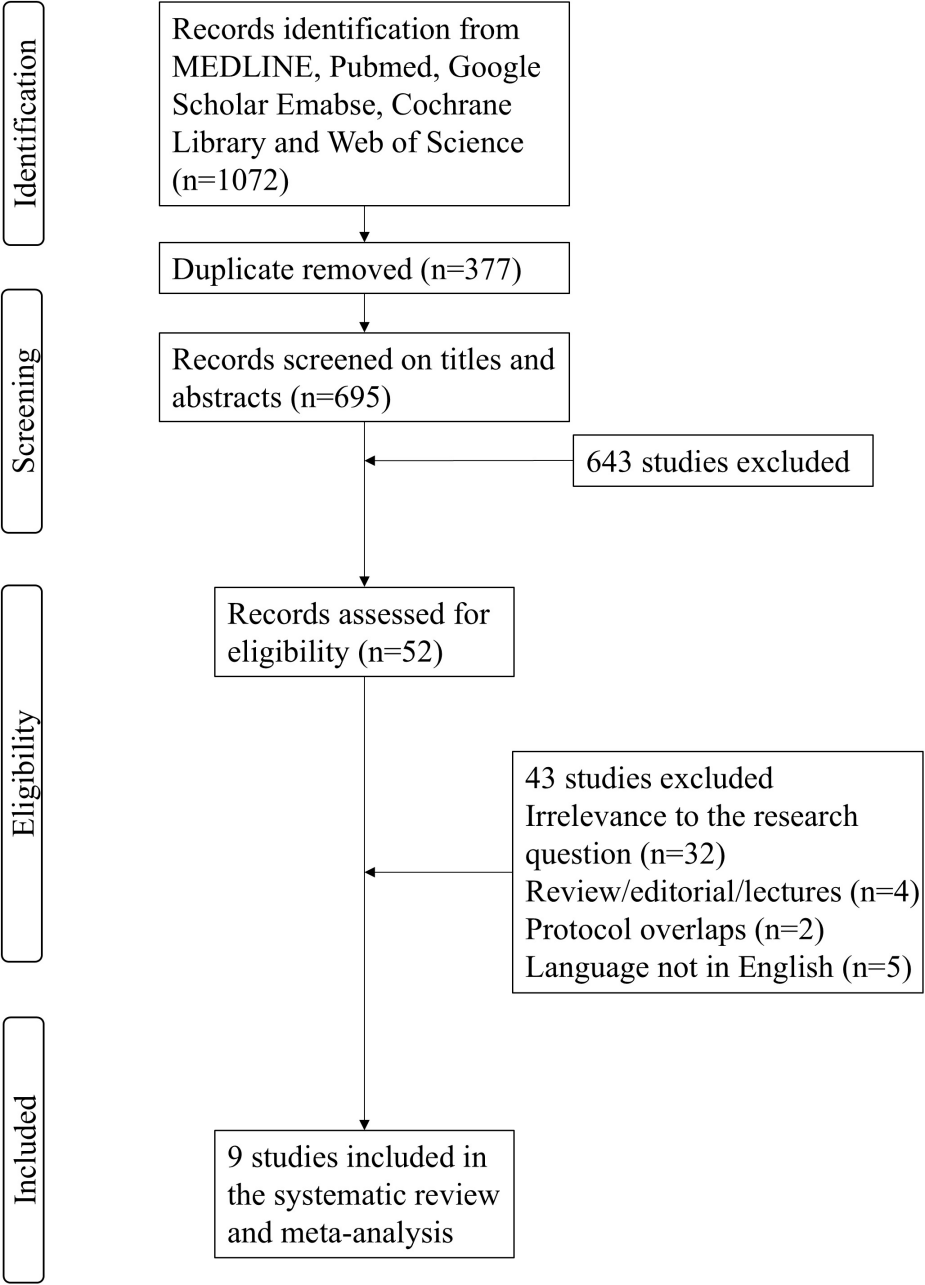
PRISMA-2020 flow diagram of search and inclusion strategy.

The systematic search identified a total of 1,072 citations from electronic databases and manual sources, with 377 duplicates removed, yielding 695 unique records for initial screening. After title and abstract review, 52 potentially relevant articles were selected for full-text evaluation. Following rigorous assessment against predefined inclusion criteria (e.g., study design, population, intervention), 43 articles were excluded due to irrelevance to the research question, insufficient data, or protocol overlaps. Ultimately, nine studies met all eligibility requirements and were included in the qualitative synthesis (Ding et al., 2018; Ge et al., 2016; Tian et al., 2016; Tian et al., 2017; Tian et al., 2020; Wu et al., 2023; Xie et al., 2021; Yang et al., 2021; Zhang et al., 2021). Of these, eight studies provided extractable outcome data for quantitative meta-analysis. The selection process adhered to PRISMA guidelines, with discrepancies resolved through consensus discussions among reviewers (Figure 1).

Table 1 summarized the characteristics of nine studies included in the meta-analysis. The sample size of included studies ranges from 8 to 85. Majority studies were single-arm studies evaluating FMT on chronic constipation (Ding et al., 2018; Ge et al., 2016; Tian et al., 2016; Tian et al., 2020; Wu et al., 2023; Xie et al., 2021; Zhang et al., 2021). One study compared FMT and biofeedback therapy on constipation (Yang et al., 2021). One RCT compared FMT and conventional therapy on constipation (Tian et al., 2017). The follow-up period ranges from 4 to 48 weeks. Four studies analyzed gut microbiota composition pre- and post-FMT (Tian et al., 2020; Xie et al., 2021; Yang et al., 2021; Zhang et al., 2021). Table 2 presents the microbiota composition data of included studies. Two studies also performed the metabolite analysis pre- and post FMT therapy (Xie et al., 2021; Zhang et al., 2021).

**TABLE 2:** Microbiota composition and metabolite data in included studies.

References	Microbiota measurement	Microbiota composition data ↑after FMT	Microbiota composition data ↓after FMT	Key beneficial bacteria	Key metabolite
Guyatt et al., 2011	N/A	N/A	N/A	N/A	N/A
Ge et al., 2016	N/A	N/A	N/A	N/A	N/A
Tian et al., 2016	N/A	N/A	N/A	N/A	N/A
Tian et al., 2017	N/A	N/A	N/A	N/A	N/A
Ding et al., 2018	Alpha and Beta diversity, LEfSe	*Prevotella*, *Megamonas*, *Acidaminococcaceae*, *Butyricimonas*	Enterobacteriaceae Bacteroides	*Prevotella*, *Megamonas*, *Acidaminococcus*, *Butyricimonas*	N/A
Tian et al., 2020	Alpha & Beta diversity, PCoA, LEfSe	*Paraprevotella*, *Weissella*, *Coprococcus*, *Phascolarctobacterium*, *Allisonella*, *Fusicatenibacter*, *Acidaminococcaceae*, *Leuconostocaceae*, *Clostridia*	Lachnoanaerobaculum, Anaerofilum, Neisseria	*Fusicatenibacter*, *Paraprevotella*	Butyric acid↑
Zhang et al., 2021	Alpha and Beta diversity, PCoA, LEfSe	Actinobacteria (*Bifidobacterium*), Proteobacteria (*Escherichia*), Firmicute (*Lactobacillus*)	Bacteroidetes (*Prevotell, Bacteroides*), Firmicute (*Roseburia, Blautia*)	*Lactobacillus*	N-Acetyl-Lglutamate, gamma-L-Glutamyl-L-glutamic acid, Glycerophosphocholine
Xie et al., 2021	Alpha and Beta diversity, PCoA,	*Prevotella*, *Bifidobacterium*, *Paraprevotella*, *Collinsella*, *Barnesiellaceae*, *Actinobacteria*, *Slackia*, *Adlercreutzia*, *Enterococcus*	Bacteroidaceae, Victivallaceae	*Prevotella*, *Bifidobacterium*, *Actinobacteria*	N/A
Yang et al., 2021	N/A	N/A	N/A	N/A	N/A

LEfSe, LDA Effect Size; PCoA, Co-ordinates Analysis.

Among the included studies, eight studies had available data to analyze the remission rate of FMT on constipation (Ding et al., 2018; Ge et al., 2016; Tian et al., 2016; Tian et al., 2017; Tian et al., 2020; Wu et al., 2023; Xie et al., 2021; Zhang et al., 2021). Seven studies had available data to analyze the improvement rate of FMT on constipation (Ding et al., 2018; Ge et al., 2016; Tian et al., 2016; Tian et al., 2017; Tian et al., 2020; Wu et al., 2023; Xie et al., 2021), seven studies (Ge et al., 2016; Tian et al., 2016; Tian et al., 2017; Tian et al., 2020; Wu et al., 2023; Yang et al., 2021; Zhang et al., 2021), three studies (Ge et al., 2016; Tian et al., 2016; Tian et al., 2017), six studies (Tian et al., 2016; Tian et al., 2017; Tian et al., 2020; Wu et al., 2023; Xie et al., 2021; Zhang et al., 2021), four studies (Ge et al., 2016; Tian et al., 2016; Xie et al., 2021; Yang et al., 2021), four studies (Ding et al., 2018; Ge et al., 2016; Yang et al., 2021; Zhang et al., 2021), three studies [(Ding et al., 2018; Tian et al., 2020; Zhang et al., 2021) had data on stool consistency, colonic transit time, Wexner score, GIQLI, PAC-SYM and PAC-QOL respectively.

### Pooled efficacy of FMT in the treatment of chronic constipation

**FIGURE 2:**
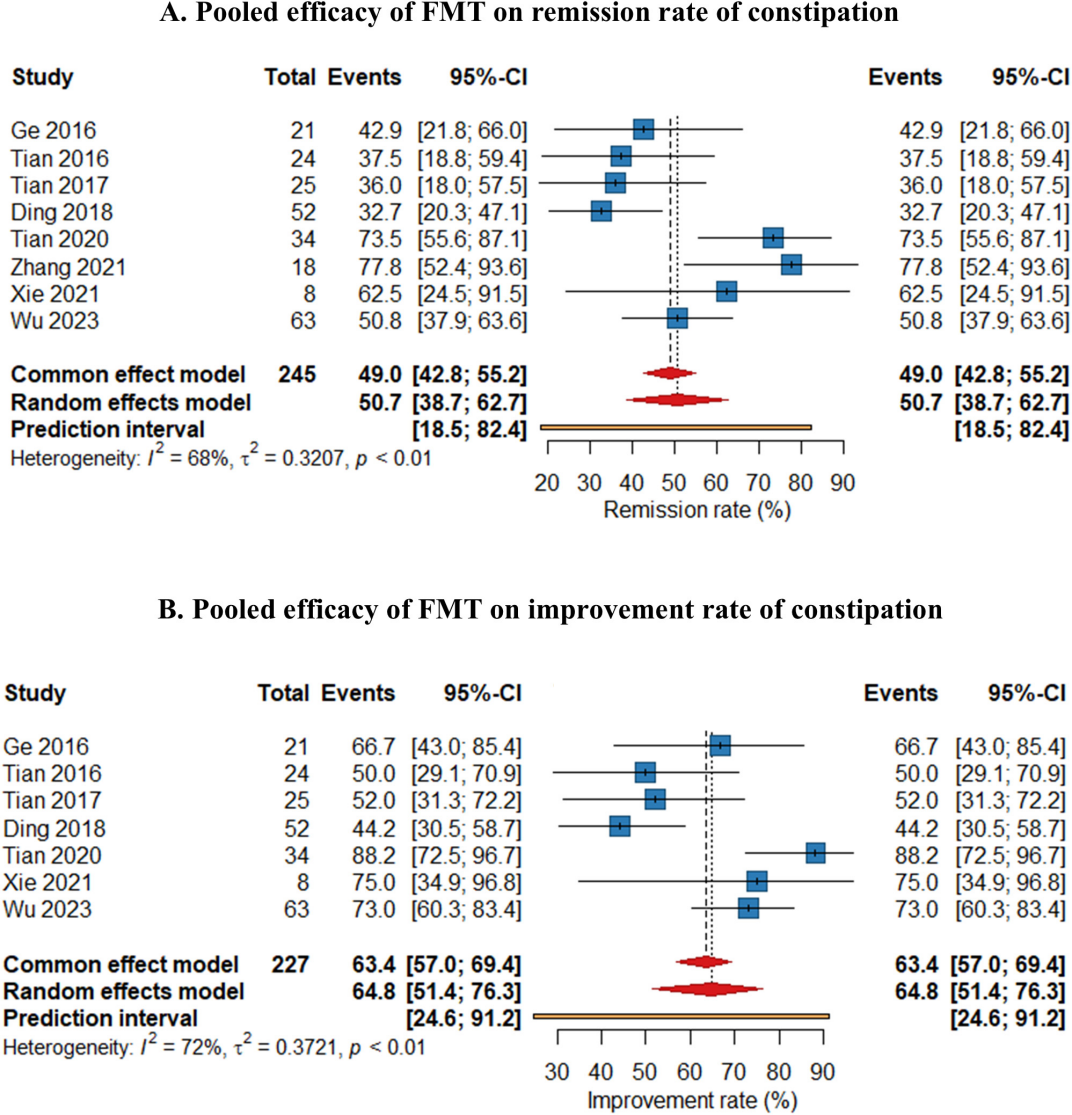
Forest plots of efficacy of fecal microbiota transplantation (FMT) on remission rate **(A)** and improvement rate **(B)** of constipation.

**FIGURE 3:**
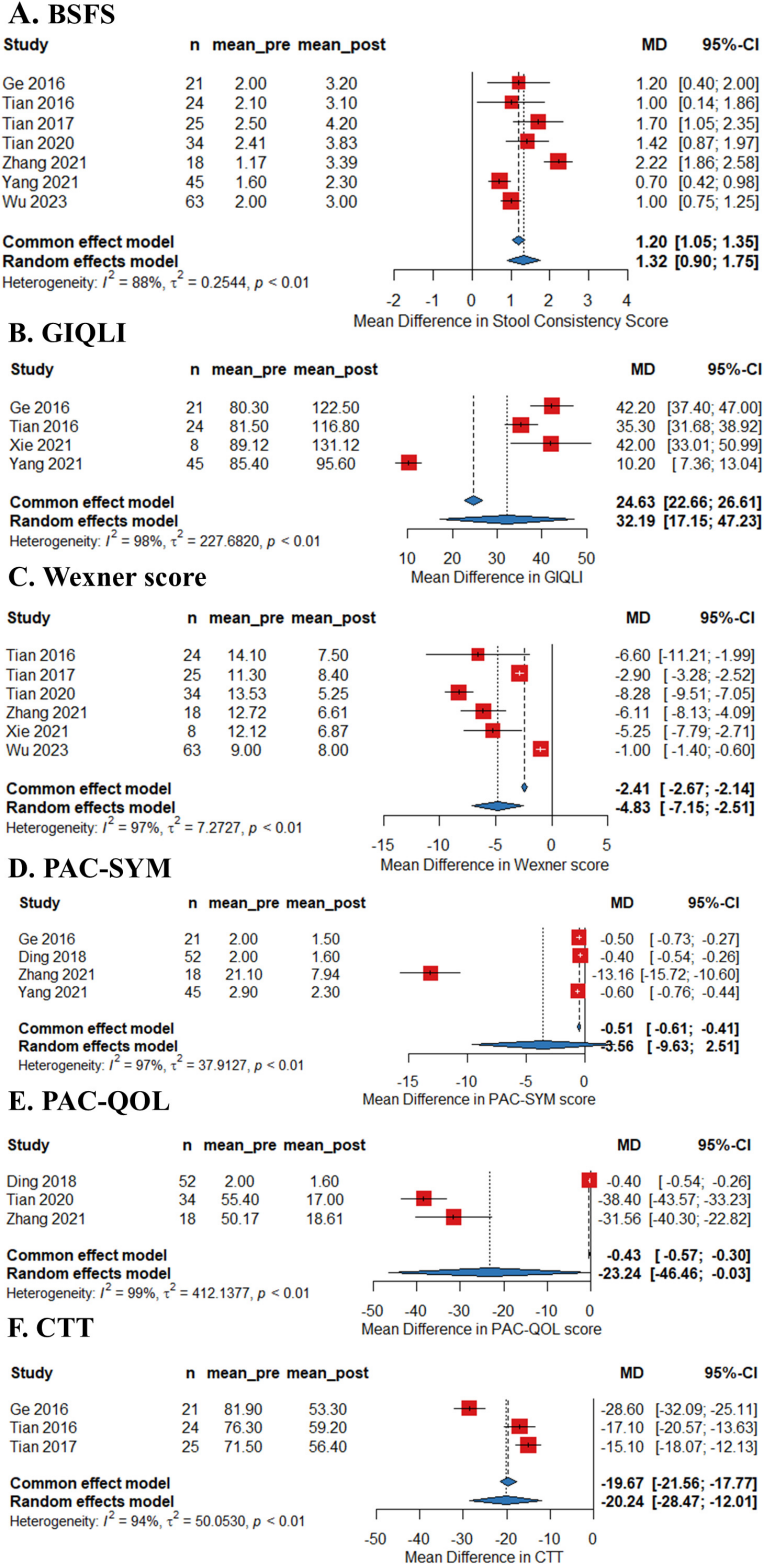
Forest plots of efficacy of fecal microbiota transplantation (FMT) on Bristol Stool Form Scale (BSFS) **(A)**, Gastrointestinal Quality-of-Life Index (GIQLI) **(B)**, Wexner score **(C)**, Patient Assessment of Constipation-Symptoms (PAC-SYM) **(D)**, and Patient Assessment of Constipation Quality of Life (PQC-QOL) **(E)** and colonic transit time (CTT) **(F)** of constipation.

As shown in Figure 2, the pooled efficacy of FMT on remission rate of constipation was 50.7% (95%CI 38.7%–62.7%) from eight studies (245 patients, I^2^ = 68%). The pooled efficacy of FMT on improvement rate of constipation was 64.8% (95% 51.4%–76.3%) from seven studies (237 patients, I^2^ = 72%), indicating that FMT may improve bowel movement in patients with chronic constipation. Meta-analysis also revealed that FMT improved stool consistency and patients’ quality of life, with pooled increase mean difference (MD) of 1.32 points (95% CI: 1.05–1.35, 230 patients, I^2^ = 88%) and 32.19 points (95%CI: 17.15–47.23, 98 patients, I^2^ = 98%) for BSFS and GIQLI score respectively. FMT also significantly relief the constipation related symptoms and improve quality of life, as shown in the pooled MD as −4.83 points (95% CI: −7.15 to 2.51, 172 patients, I^2^ = 97%), −3.56 points (95% CI: −9.63 to 2.51, 136 patients, I^2^ = 96.4%), −23.2 points (95% CI: −46.5 to 0.03, 104 patients, I^2^ = 99%) for Wexner constipation scale, PAC-SYM and PAC-QOL, respectively. In addition, colonic transit time was also significantly decreased after FMT treatment, as shown in the pooled decrease of MD as −20.3 h (95% CI: −28.5 to 12.0 h, I^2^ = 94%) (70 patients) from three studies (Figures 3A–F). The summary of the meta-analysis was present in Table 3.

**TABLE 3:** Summary of findings and certainty of the evidence.

Outcomes	Measurement	Studies (participants)	Pooled efficacy of FMT (95% CI, 95% PI)	Heterogeneity (Q, *p*-value, I^2^, %)	Certainty of the evidence (GRADE)*	Conclusion
Remission rate	CSBM	8 studies (245) (Ding et al., 2018; Ge et al., 2016; Guyatt et al., 2011; Tian et al., 2016; Tian et al., 2017; Tian et al., 2020; Yang et al., 2021; Zhang et al., 2021)	50.7% (38.7–62.7%) (18.5–82.4%)	Substantial (26, < 0.05, 68%)	Very low 1, 2, 3	Approximately half of patients achieved clinically completely symptom relief by FMT therapy
Improvement rate	CSBM	7 studies (237) (Ding et al., 2018; Ge et al., 2016; Guyatt et al., 2011; Tian et al., 2016; Tian et al., 2017; Yang et al., 2021; Zhang et al., 2021)	64.8% (51.4–76.3%) (24.6–91.2%)	Substantial (18.9, < 0.05, 72%)	Very low 1, 2, 3	64.8% of patients achieved clinical improvement of symptom relief by FMT therapy
Stool consistency	BSFS	7 studies (230) (Ding et al., 2018; Ge et al., 2016; Guyatt et al., 2011; Tian et al., 2016; Tian et al., 2020; Xie et al., 2021; Yang et al., 2021)	1.32 (1.05–1.35)	Considerable (32.1, < 0.05, 88%)	Very low, 1, 2, 4	FMT significantly improved stool consistency scores by a mean difference of 1.32 points.
Constipation related quality of life	GIQLI	4 studies (98) (Ge et al., 2016; Guyatt et al., 2011; Xie et al., 2021; Zhang et al., 2021)	32.19 points (17.15–47.23)	Considerable (96°3, < 0.05, 98%)	Very low 1, 2, 4, 5	The pooled MD of 32.19 points exceeds the minimally clinically important difference for GIQLI (typically 10–15 points), indicating substantial improvement in gastrointestinal quality of life post-FMT.
	Wexner score	6 studies (172) (Ding et al., 2018; Ge et al., 2016; Tian et al., 2016; Tian et al., 2020; Yang et al., 2021; Zhang et al., 2021)	−4.83 points (−7.15 to 2.51)	Considerable (38.7, < 0.05, 97%)	Very low 1, 2, 4, 5	FMT significantly reduced Wexner scores by 4.83 points, indicating improved constipation symptoms
	PAC-SYM	4 studies (136) (Guyatt et al., 2011; Tian et al., 2017; Tian et al., 2020; Xie et al., 2021)	−3.56 points (−9.63 to 2.51)	Considerable (83.1, < 0.05, 96.4%)	Very low 1, 2, 3, 5	The pooled MD of −3.56 points suggested a non-significant trend toward symptom improvement (*p* = 0.37) of FMT.
	PAC-QOL	3 studies (104) (Ding et al., 2018; Tian et al., 2017; Tian et al., 2020)	−23.2 points (−46.5 to 0.03)	Considerable (54.8, < 0.05, 99%)	Very low 1, 2, 4, 5	The pooled MD of −23.2 points suggested a statistically significant improvement in PAC-QOL scores post-FMT (*p* = 0.002).
Bowel movement	CTT	3 studies (70) (Ge et al., 2016; Guyatt et al., 2011; Tian et al., 2016)	−20.3 h (−28.5 to 12.0)	Considerable (50.1, < 0.05, 94%)	Very low 1, 2, 4, 5	FMT significantly reduced CTT by 20.3 h (95% CI: −28.5 to −12.0), demonstrating accelerated colonic transit.

*(1). Downgraded one point because of moderate or high risk of bias associated with study attrition, prognostic factor measurement, and statistical analysis. (2). Downgraded one point because of inconsistency due to substantial heterogeneity. (3). Downgraded one point because of publication bias. (4). Downgraded one point because of inconsistency of forest plot. (5). Downgraded one point because of imprecision. FMT, fecal microbiome transplantation; CSBM, complete weekly spontaneous bowel movements; CTT, colonic transit time; BSFS, Bristol Stool Form Scale; PAC-SYM, Patient Assessment of Constipation-Symptoms; SBMs: spontaneous bowel movements; PAC-QOL, Patient Assessment of Constipation Quality of Life, GIQLI, Gastrointestinal Quality-of-Life Index.

### Dynamics of gut microbiome after FMT treatment

Four studies performed fecal microbiome testing based on 16S rDNA Gene analysis before and after FMT of the included patients. All four studies revealed that the composition of fecal microbiota in the post-FMT patients significantly differed from baseline in the phylum-level and genus-level abundances. Alpha and Beta diversity analysis also revealed that gut microbiota diversity elevated in patients after FMT Treatment.

As summarized in Table 2, following FMT, the bacterial taxa demonstrating significant enrichment predominantly included Phylum Firmicutes (included *Coprococcus*, *Phascolarctobacterium*, *Acidaminococcaceae, Lactobacillus, Megamonas, Fusicatenibacter*); Phylum Bacteroidetes (included *Prevotella*, *Paraprevotella*, *Butyricimonas*); Phylum Actinobacteria (included *Bifidobacterium, Collinsella, Slackia*); Phylum Proteobacteria (included *Escherichia)* and Other Notable Taxa (included *Weissella and Allisonella*).

fecal microbiota transplantation led to consistent reductions in bacterial taxa associated with pro-inflammatory or dysbiosis-related functions, categorized as follows: Phylum Proteobacteria (included *Enterobacteriaceae and Neisseria*); Phylum Bacteroidetes (included *Bacteroidaceae*); Phylum Firmicutes (included *Lachnoanaerobaculum, Anaerofilum, Roseburia* and *Blautia*); Phylum Lentisphaerae (*Victivallaceae*).

The critical bacterial taxa linked to the therapeutic effects of FMT on chronic constipation include phylogenetically and functionally distinct groups: Phylum Bacteroidetes (included *Prevotella, Paraprevotella and Butyricimonas*); Phylum Firmicutes (*Megamonas, Fusicatenibacter and Lactobacillus*); Phylum Actinobacteria (*Bifidobacterium and Actinobacteria*) and Family Acidaminococcaceae (*Acidaminococcus*) (Table 2).

### Safety of FMT

The safety of FMT was rigorously evaluated across nine included studies, with seven explicitly documenting treatment-related adverse events (AEs) (Ding et al., 2018; Ge et al., 2016; Tian et al., 2017; Tian et al., 2020; Wu et al., 2023; Xie et al., 2021; Yang et al., 2021). Critically, no severe AEs (e.g., systemic infections, hospitalization, or mortality) were reported in any studies. The majority of AEs were transient, mild-to-moderate gastrointestinal symptoms, including diarrhea (0.9%–9.5%), abdominal pain (0.9%–14.3%), flatulence (1.8%–17.3%), nausea (0.9%–6.7%), and vomiting (0%–9.5%), all of which resolved spontaneously without intervention. A subset of studies identified procedure-related discomfort, such as naso-intestinal tube irritation, that ceased immediately post-FMT. Mechanistically, these observations align with the transient microbial engraftment dynamics and procedural tolerability. Collectively, the absence of severe AEs and the self-limiting nature of reported symptoms underscore the favorable safety profile of FMT in functional constipation, supporting its feasibility for further clinical translation.

### Quality assessment and heterogeneity analysis

The methodological rigor of included studies was evaluated using domain-specific tools. For Tian’s study, RoB 2 tool revealed critical concerns: high risk in outcome measurement due to unblinded assessment (patients are unblined). Observational studies (*n* = 8) scored fair on the Newcastle-Ottawa Scale (NOS). Common limitations included insufficient adjustment for confounding variables (e.g., laxative use or not) and non-representative sampling of constipated populations. Detailed risk of bias assessments is provided in Supplementary Tables 1, 2.

We also performed sensitivity analyses and the results demonstrated that the pooled remission and improvement rates remained stable with sequential exclusion of individual studies, indicating the overall robustness of primary findings. Nevertheless, high heterogeneity persisted across all outcomes (I^2^ > 70%), likely attributable to inter-study variations in donor screening strategies (e.g., inclusion/exclusion of constipated donors), fecal preparation methods (fresh vs. frozen), and differential follow-up periods.

## Discussion

In this systematic review and meta-analysis, we demonstrate that fecal microbiota transplantation (FMT) holds significant potential to alleviate chronic constipation. Our synthesis of nine clinical studies reveals that FMT achieved complete symptom remission in 50.7% of patients and measurable improvement in 66.7%, with parallel enhancements in stool consistency, colonic transit time, and quality-of-life metrics. Mechanistically, four integrated microbiota analyses identified a post-FMT expansion of phylogenetically and functionally distinct taxa: *Prevotella and Paraprevotella* (Bacteroidetes)–polysaccharide degraders linked to mucosal glycoprotein metabolism; *Fusicatenibacter and Megamonas* (Firmicutes)–butyrate producers critical for enteric neuronal activation; *Bifidobacterium* (Actinobacteria)–a keystone genus modulating gut motility through acetate-mediated vagal signaling; and *Acidaminococcus* (Acidaminococcaceae), a glutamate-fermenting symbiont implicated in visceral hypersensitivity regulation. These taxonomic shifts align with preclinical models where microbial-derived metabolites (e.g., butyrate, acetate) enhance neuromuscular coordination and dampen proinflammatory cascades. Despite transient gastrointestinal symptoms in some of the recipients, the absence of severe adverse events underscores FMT’s short-term safety. However, heterogeneity in donor selection, delivery routes, and remission criteria complicates cross-study comparisons, while the lack of randomized controlled trials and long-term data precludes causal inference.

The therapeutic landscape for chronic constipation reveals striking contrasts in efficacy across interventions. Conventional first-line therapies—osmotic laxatives like polyethylene glycol (PEG) and stimulants such as bisacodyl–demonstrate 30%–50% response rates in clinical trials, with high relapse rates upon discontinuation (Chassagne et al., 2017; Luthra et al., 2019; Nelson et al., 2017). Probiotics show modest symptom improvement (15%–25% over placebo) in meta-analyses, though strain-specific effects limit generalizability (Dimidi et al., 2020; Huang et al., 2025). Biofeedback therapy achieves 45%–55% sustained response in pelvic floor dysfunction subtypes yet requires specialized infrastructure (Chiarioni et al., 2006; Rao et al., 2010). Against this backdrop, FMT’s 50.7% remission rate (95% CI: 38.7%–62.7%) and 66.7% improvement rate in refractory cases–derived from eight studies of 245 patients–suggests microbiome modulation outperforms these approaches in specific populations. Crucially, FMT concurrently elevates stool consistency (BSFS MD = 1.32) and gastrointestinal quality of life (GIQLI MD = 32.19), outcomes rarely achieved by monotherapies. Four studies (Tian et al., 2020; Xie et al., 2021; Yang et al., 2021; Zhang et al., 2021) trace these clinical gains to durable microbiota restructuring, while two (Xie et al., 2021; Zhang et al., 2021) implicate bile acid and short-chain fatty acid metabolic shifts–mechanistic dimensions absent in conventional interventions. This biosynthetic capacity positions FMT not merely as a symptomatic treatment, but as an ecosystem-level reset for dysbiotic constipation phenotypes. Variability in preparation methods (fresh vs. frozen), delivery routes (colonoscopic vs. oral), and dosing schedules obscures causal links between microbial signatures and clinical outcomes–a challenge mirrored across FMT applications. For FMT therapy in most clinical studies, currently no studies have directly compared the efficacy of different FMT delivery routes (e.g., colonoscopic vs. oral capsules). In inflammatory bowel disease (IBD), current evidence suggests no significant differences in clinical outcomes, such as mucosal healing rates, have been observed between fresh and frozen FMT preparations (Fehily et al., 2021). Among the 9 studies included in our analysis, all FMT interventions for constipation utilized nasoenteric tube administration. Consequently, there is a lack of evidence directly comparing the efficacy differences between various delivery routes (e.g., nasoenteric tube vs. oral or colonoscopic approaches). Current data remains insufficient to clarify which administration route holds a definitive advantage. Standardization must prioritize donor selection criteria and engraftment monitoring to isolate therapeutic strains and metabolites.

Across nine studies in this systematic review, systematic adverse (AE) tracking identified only transient gastrointestinal disturbances–diarrhea (0.9%–9.5%), abdominal pain (9%–14.3%), flatulence (1.8%–17.3%) –and minor procedure-linked effects (e.g., naso-intestinal irritation) that resolved without intervention (Ge et al., 2016; Tian et al., 2017; Wu et al., 2023; Xie et al., 2021; Yang et al., 2021). Rigorous donor screening protocols likely contributed to the absence of severe infections or systemic complications, reinforcing methodological priorities in microbial therapeutic development. Mechanistic parallels between these self- and transient post-FMT microbial engraftment dynamics invite deeper exploration of-microbial adaptability. This collective safety evidence, anchored in adverse resolution and procedural robustness, elevates FMT clinical feasibility. Nevertheless, targeted studies must now address granular risk-benefit stratification, including donor-recipient strain compatibility, to propel rational translation.

Post-FMT microbial profiling across four studies (Tian et al., 2020; Xie et al., 2021; Yang et al., 2021; Zhang et al., 2021) revealed a functional restructuring of gut communities, marked by enrichment of phylogenetically distinct taxa with motility-modulating and anti-inflammatory properties. Recipients exhibited increased abundance of *Prevotella* and *Paraprevotella* (Bacteroidetes)–mucin specialists linked to enhanced mucosal glycoprotein turnover (Glover et al., 2022) –and *Megamonas* and *Fusicatenibacter* (Firmicutes), which drive butyrate synthesis to stimulate enteric neurons (Cavaliere and Traina, 2023; Lanza et al., 2021). Concurrently, FMT suppressed pro-inflammatory *Enterobacteriaceae* (Proteobacteria) and *Bacteroidaceae*, taxa associated with gut barrier disruption and visceral hypersensitivity. Critically, *Bifidobacterium* (Actinobacteria) and *Acidaminococcus* (Acidaminococcaceae) emerged as keystone taxa, their post-FMT expansion correlating with acetate-mediated vagal signaling and glutamate metabolism, respectively (Mayer et al., 2015; Zhang et al., 2022). These shifts coincided with elevated alpha diversity and restored beta diversity patterns, suggesting FMT reestablishes ecological resilience in constipated microbiomes.

The taxonomic and functional shifts above may align with a multi-hit mechanism for FMT efficacy: (1) butyrate-driven neuromuscular activation via Firmicutes (*Fusicatenibacter*) restores colonic motility (Lanza et al., 2021; Tixier et al., 2006); (2) acetate and GABA modulation by *Bifidobacterium* and *Acidaminococcus* dampens visceral pain signaling (Duranti et al., 2020; Pokusaeva et al., 2017); (3) mucosal barrier repair mediated by *Prevotella*-derived mucin degradation reduces endotoxin translocation (Wright et al., 2000; Yuan et al., 2022). These findings nominate *Prevotella* and *Bifidobacterium* abundance and *Enterobacteriaceae* suppression as candidate biomarkers for FMT responsiveness. Yet, causal validation requires integrating metagenomics, metabolomics, and host transcriptomics in longitudinal cohorts to disentangle microbial contributions from confounders like diet or transit time. Standardizing donor selection around these taxa–while prioritizing butyrogenic consortia–may optimize FMT’s therapeutic precision.

Our synthesis integrates clinical and microbiome data across nine studies (Ding et al., 2018; Ge et al., 2016; Tian et al., 2016; Tian et al., 2017; Tian et al., 2020; Wu et al., 2023; Xie et al., 2021; Yang et al., 2021; Zhang et al., 2021), offering the first holistic assessment of FMT’s dual role in alleviating chronic constipation and restructuring gut ecosystems. The substantial heterogeneity observed across clinical outcomes (I^2^ = 72.9%–96.4%) reflects both methodological diversity and biological complexity in FMT interventions. While our synthesis adhered to PRISMA guidelines and employed random-effects models to account for variability, the interpretative power remains constrained by inherent limitations: (1) Protocol heterogeneity–divergent donor screening practices (e.g., only 33% of studies excluded constipated donors), fecal processing methods (fresh vs. frozen), and remission criteria; (2) Temporal discordance–follow-up durations ranging from 4 to 48 weeks, with diminishing response rates beyond 12 weeks suggesting transient engraftment; (3) Ecological compatibility gaps–unstandardized donor-recipient matching potentially compromising motility-regulating taxa (e.g., *Bifidobacterium*) survival. Despite these challenges, the convergence of multi-omics evidence (Xie et al., 2021; Zhang et al., 2021)—linking stool consistency improvements (MD = 0.94, 95% CI: 0.42–1.46) to *Bifidobacterium*-driven metabolite shifts—strengthens causal inference for FMT’s pleiotropic mechanisms.

The lack of control groups in most studies precludes definitive attribution of symptom improvement to FMT itself, as placebo effects—which account for 30%–40% of response in functional gastrointestinal disorders—cannot be ruled out. While our pooled estimates suggest clinical potential, the open-label designs inherently conflate microbiota-mediated effects with natural disease fluctuation and non-specific therapeutic expectations. Furthermore, the Tian et al. (2017) RCT’s active comparator design, while pragmatic for clinical practice, inherently conflates FMT efficacy with relative superiority over laxatives rather than absolute therapeutic mechanisms. In addition, publication bias likely inflates the observed 50.5% remission rate. Furthermore, microbiota meta-analyses were hampered by non-standardized reporting of diversity metrics (e.g., conflicting definitions of alpha diversity) and taxonomic classification (e.g., *Prevotell* labeled as beneficial or pathogenic across studies). Quality appraisal revealed methodological limitations in included RCTs. Tian’s et al. (2017) exhibited high risk in outcome measurement due to unblinded design—a recognized challenge in FMT trials given the procedural distinctiveness. While observational studies demonstrated adequate selection criteria, the lack of adjustment for laxative use in 6/7 cohort studies may confound efficacy estimates.

The predominance of single-arm studies (8/9 trials) and inclusion of only one underpowered RCT markedly constrain causal inference. While our pooled analyses suggest therapeutic potential, the current evidence level parallels Phase II exploratory trials, necessitating validation through large-scale, double-blind RCTs with sham-FMT controls. Future research must prioritize adequately powered RCTs employing standardized protocols to isolate microbiota-specific effects from placebo responses. Such trials should integrate longitudinal multi-omics (e.g., metagenomics, metabolomics) to mechanistically dissect donor-recipient interactions—a prerequisite for advancing FMT from empirical practice to precision microbial therapeutics. This requires harmonized frameworks integrating three pillars: (1) rigorous donor screening through metagenomic profiling to exclude dysbiotic signatures (e.g., methanogen overgrowth); (2) longitudinal multi-omics tracking (> 24 weeks) to differentiate durable microbial engraftment from transient ecological shifts; and (3) mechanistic validation of microbiota-derived metabolites (e.g., butyrate) through parallel gnotobiotic models. Only through such coordinated efforts can we transform FMT from empirical practice into targeted ecosystem therapeutics, ensuring sustained clinical benefits beyond temporary microbiota perturbations.

## Conclusion

This systematic review establishes fecal microbiota transplantation (FMT) as a mechanistically grounded therapy for chronic constipation, demonstrating clinically meaningful symptom remission (50.7%) coupled with functional restructuring of gut ecosystems—specifically enriching *Bifidobacterium* and butyrate-producing *Fusicatenibacter* while suppressing pro-inflammatory *Enterobacteriaceae*. These microbial shifts may correlate with restored gut-brain axis signaling through metabolites like butyrate and acetate, which enhance neuromuscular coordination and barrier integrity. While rodent models provide mechanistic hypotheses (e.g., butyrate’s role in serotonin synthesis), the extrapolation to human pathophysiology remains provisional. Concurrent human host-response data—such as colonic serotonin receptor expression or fecal metabolomics—are critically needed to confirm causality.

In summary, current evidence remains provisional due to heterogeneous protocols, transient engraftment, and undersized cohorts. To transcend empirical application, future RCTs must prioritize standardized donor-recipient matching, longitudinal multi-omics profiling (> 24 weeks), and mechanistic validation of microbial metabolites in gnotobiotic models. Success in these areas will propel FMT into precision microbiome engineering—a paradigm that recalibrates gut ecology at the ecosystem level, addressing refractory constipation through sustained microbiota-host dialogue rather than symptomatic relief alone.

## Data Availability

The raw data supporting the conclusions of this article will be made available by the authors, without undue reservation.
