# Supplementary material for: Fecal microbiota transplantation for chronic constipation: a systematic review and meta-analysis of clinical efficacy, safety, and microbial dynamics
Source: Front Microbiol. 2025 Jul 31;16:1604571. doi: 10.3389/fmicb.2025.1604571 (PMC12350361; doi:10.3389/fmicb.2025.1604571)
Supplement: Supplementary file 1 [file Table_1.docx]

Supplemental Table 1. Newcastle Ottawa Quality Assessment for the Included Studies

| Author (ref) | 1-Representativeness of the Exposed Cohort? *=Truly or somewhat representative | 1-Selection of the non-exposed? *= Drawn from the same community | 1-Ascertainment of exposure? *=Secure record or structured interview | 1-Demonstration That outcome was not present at the start of the study? *= Yes | 2-Comparability based on design or analysis controlled for confounders *=Yes | 3-Assessment of outcome. *=Independent assessment or record linkage | 3-Was follow-up long enough for outcomes to occur? *=Yes | 3-Adequacy of follow-up of cohorts *=No loss of follow-up of patients (No attrition bias) | Result |
| --- | --- | --- | --- | --- | --- | --- | --- | --- | --- |
| Ge ^[25]^ | * |  | * | * | * | * | * |  | Fair |
| Tian ^[26]^ | * |  | * | * | * | * | * | * | Good |
| Ding ^[28]^ | * |  | * | * |  | * | * | * | Fair |
| Tian ^[29]^ | * |  | * | * | * | * | * |  | Fair |
| Zhang ^[30]^ | * |  | * | * | * | * |  |  | Fair |
| Xie ^[31]^ | * |  | * |  | * | * |  |  | Fair |
| Yang ^[32]^ | * | * | * | * | * | * |  | * | Good |
| Wu ^[33]^ | * |  | * | * | * | * | * | * | Good |

(1=Selection domain, 2=Comparability domain, 3=Outcome/exposure domain. Good quality: 3 or 4 stars in selection domain AND 1 star in comparability domain AND 2 or 3 stars in outcome/exposure domain; Fair quality: 2 stars in selection domain AND/OR 0 or1 stars in comparability domain AND 2 or 3 stars in outcome/exposure domain; Poor quality: 0 or 1 star in selection domain OR 0 stars in comparability domain OR 0 or 1 stars in outcome/exposure.)
