# Supplementary material for: Fecal microbiota transplantation for chronic constipation: a systematic review and meta-analysis of clinical efficacy, safety, and microbial dynamics
Source: Front Microbiol. 2025 Jul 31;16:1604571. doi: 10.3389/fmicb.2025.1604571 (PMC12350361; doi:10.3389/fmicb.2025.1604571)
Supplement: Supplementary file 2 [file Table_2.docx]

Supplemental Table 2. Risk of bias assessment for RCT

| Study ^(ref)^ | Randomization | Deviations | Missing data | Measurement | Reporting | Overall |
| --- | --- | --- | --- | --- | --- | --- |
| Tian ^[27]^ | Some concerns | Some concerns | Low risk | Low risk | Some concerns | Some concerns |

(Bias domain and signalling question: Ransomization--Bias arising from the randomisation process; Deviations--Bias due to deviations from intended interventions; Missing data--Bias due to missing outcome data; Measurement--Bias in measurement of the outcome; Reporting-- Bias in selection of the reported result; Overall--Overall bias.)
